# Developing Digital Home Assistant User Guides for Older Adults With and Without Long-Term Mobility Disabilities

**DOI:** 10.1093/geroni/igaa057.2220

**Published:** 2020-12-16

**Authors:** Travis Kadylak, Kenneth Blocker, Widya Ramadhani, Lyndsie Koon, Roshanak Khaleghi, Chris Kovac, Ramavarapu Sreenivas, Wendy Rogers

**Affiliations:** 1 University of Illinois at Urbana-Champaign, Champaign, Illinois, United States; 2 University of Illinois at Urbana-Champaign, Urbana, Illinois, United States; 3 University of Kansas, Lawrence, Kansas, United States

## Abstract

The aim of the current study was to understand how to integrate digital home assistant technologies and smart appliances into older adults’ homes by developing supportive user guides that facilitate adoption and continued use. We conducted a series of interviews among older adults, with and without mobility disabilities, to understand their attitudes towards digital assistants and to identify needs for instructional support and user guides. Subsequently, we developed and tested specific user guide modules for older adults aimed at addressing the identified concerns and desired instructional support. Specifically, we developed and field-tested user guides for the initial Amazon Echo device setup, basic device use (e.g., playing music and checking the weather), and separate modules for other domestic use cases (e.g., how to pair an Alexa enabled device with smart lights or appliances). Our results provide guidance for implementation of smart voice technologies to support older adults with long-term mobility disabilities.

